# Supplementary material for: Effect of Degradation in Small Intestinal Fluids on Mechanical Properties of Polycaprolactone and Poly-l-lactide-co-caprolactone
Source: Polymers (Basel). 2023 Jul 6;15(13):2964. doi: 10.3390/polym15132964 (PMC10347225; doi:10.3390/polym15132964)
Supplement: Supplementary file 1 [file polymers-15-02964-s001.zip › polymers-2482773-supplementary.pdf]

Supplementary Materials

Effect of Degradation in Small Intestinal Fluids on Mechanical Properties of Polycaprolactone and Poly-*L*-Lactide-*co*-Caprolactone

Sam Peerlinck, Marc Miserez, Dominiek Reynaerts and Benjamin Gorissen*

E-mail: benjamin.gorissen@kuleuven.be

This PDF file includes:

Figs. S1 to S5

Tables S1 to S3

References for SI reference citations

S1. Additional information

This section details some methods used in the determination of the degradation time-dependent mechanical properties refered to in the main text.

A. Dogbone tensile sample dimensions

Figure S1 displays the dimensions of the dogbone sample injection molding die. The depth of the die was 1 mm. Polycaprolactone (PCL) samples retained their original dimensions after molding and subsequent cooling, so their tested dimensions correspond with figure S1. Poly-*L*-Lactide-*co*-Caprolactone (PLCL) samples contracted in their longitudinal direction, resulting in a reduced section length of 13.8 mm (instead of 16 mm) and expanded in reduced section width (1.76 mm instead of 1.6 mm) and thickness (1.08 mm instead of 1 mm). This is expected to be due to material shrinking and the injection direction of the melted material, which was in the longitudinal direction. This unexpected behavior caused the reduced section length-to-width ratio to be lower than 10, possibly influencing translation of the displacement of the clamped parts to deformation of the reduced section. However, this is not problematic as the reduced section deformation was measured directly through optical data acquisition.

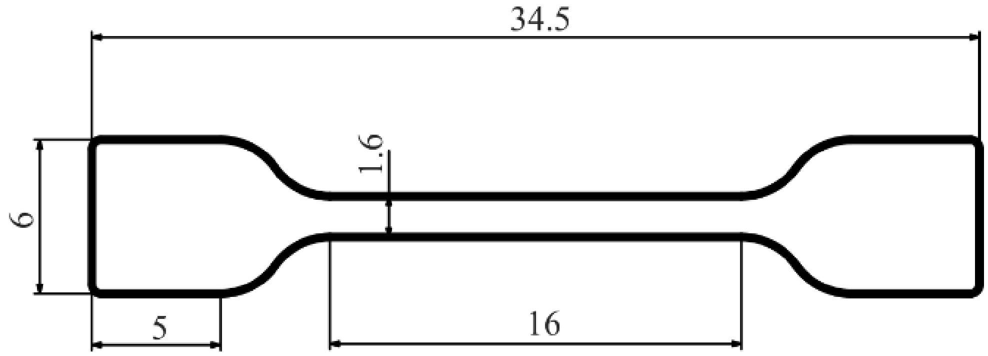

Figure S1: Dimensions of the dogbone sample injection molding die. Depth of the die is 1 mm.

B. Perfusion system for sample submersion

Figure S2 gives a schematic overview of the perfusion system refreshing the small intestinal fluids used for the degradation of the PCL and PLCL tensile testing samples. The degradation occurred at 37°C, limiting the timespan of enzymatic activity in the fluids. To ensure this enzymatic activity an automatic periodic perfusion system was constructed, with the following working procedure. Fresh fluids (**1**), collected weekly from the university hospital and with MES-buffer (pH 6.5, *Sigma-Aldrich M3671*) at 25 mMolar and Antibiotic-Antimycotic (*Life Sciences 15240062*) at 3 vol% added for stability, were stored in a refrigerator (**2**) at 4°C. The tensile samples were submerged in 50 mL centrifuge tubes (*Fisher Scientific*) (**3**), placed on a hotplate (*Velp Scientifica*) (**4**) and under an insulated hood (**5**), keeping a constant temperature of 37°C, which was periodically verified using a thermocouple. Every 24 hours, the following fluid replacement protocol was executed by an Arduino Uno microcontroller (**6**). Firstly the draining 2/2 solenoid valve (*SMC VX222EZ2AB*) (**7**) was opened for a time sufficient to empty the centrifuge tubes to the downstream waste container (**8**). Then the DC peristaltic (*Verderflex*) pump (**9**) pumped the predetermined volume of fresh intestinal fluids into the centrifuge tubes and the process was set to repeat in 24 hours. To avoid potential spillage of small intestinal fluids from the set-up, an

overflow line (**10**) was installed. If the volume would fail to drain, but the pump started regardless, the excess fluid would be evacuated through holes and tubes from the top of the centrifuge tubes.

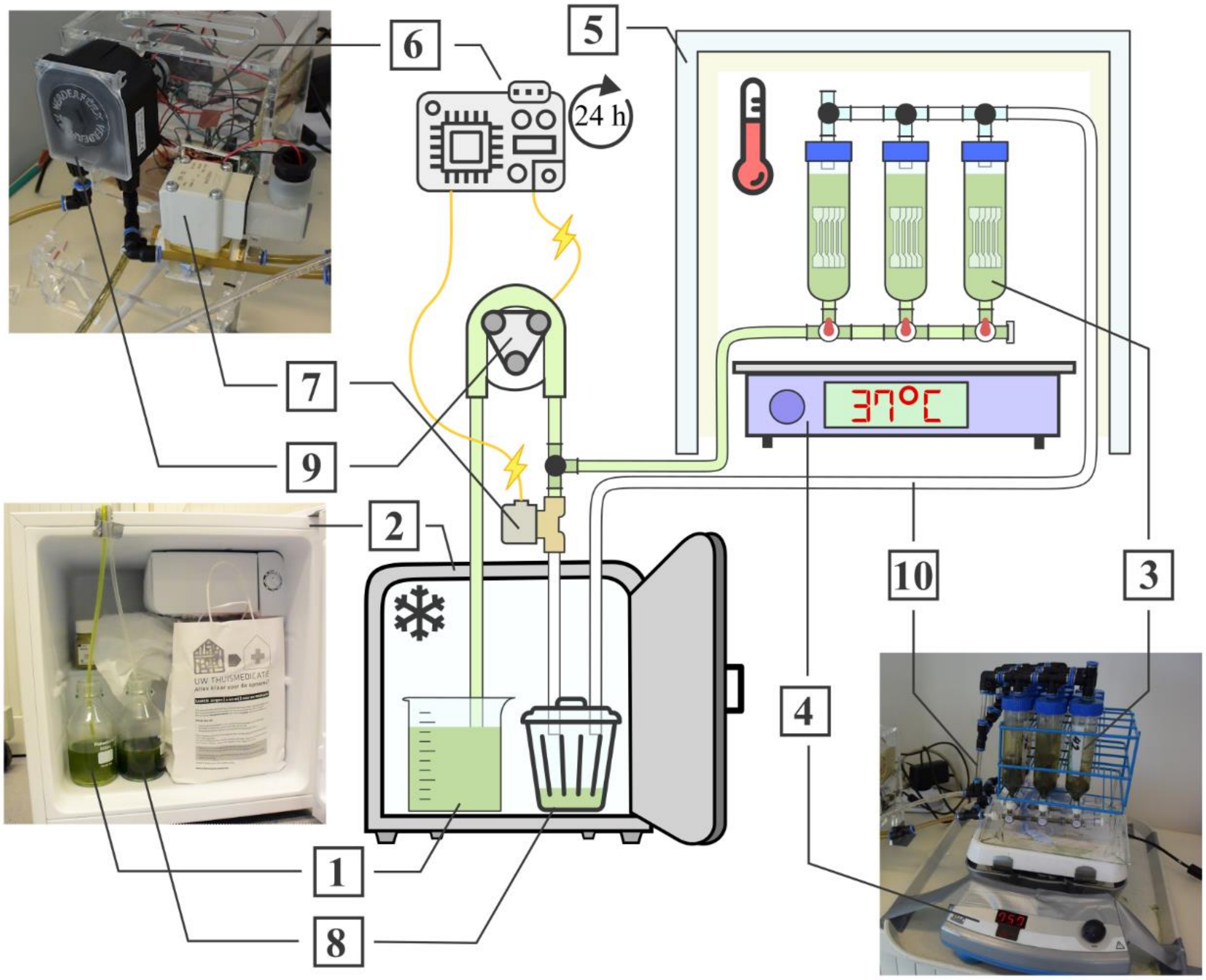

Figure S2: Schematic overview of the perfusion system for refreshing the small intestinal fluids every 24h to ensure the enzymatic activity at a temperature of 37°C.

C. Calculation of sample deformation from encoder data.

To confirm the deformation of a sample obtained from optical data, the deformation based on the data acquired from the encoders in the linear motors is calculated. Figure S3 shows the important components of the tensile testing machine, with the sample (**1**) fixated by two clamps (**2**) which are in turn connected to the load cells (**6**) and the linear motors (**4**) by two 3D-printed arms (**3**). Figure S3 D depicts the linearized model of the compliant connection between the sample of interest and the rigid load cell/motor components. When, as illustrated in figure S3 C, the linear motor imposes a displacement D_{motor}, the connecting arms deform substantially, resulting in a lower displacement of the fixating clamps D_{clamp}. The deformation of the 3D-printed arms occurs in both in the vertical plane (illustrated in figure S3 C) and the horizontal plane, and is the result of a bending moment in horizontal beam. By determining the equivalent stiffness k_{arm} of the arms, and using hooke's law on the total

deformation and the force measurements from the load cells, the deformation of the sample ε_{sample} can be estimated:

$$\varepsilon_{sample} = \frac{\Delta L_{sample}}{L_{sample,1}} = \frac{\Delta L_{total} - 2\Delta L_{arm}}{L_{sample,1}} = \frac{\Delta L_{total} - 2F/k_{arm}}{L_{sample,1}} \quad [1]$$

With $\Delta L_{sample} = L_{sample,2} - L_{sample,1}$ and $\Delta L_{total} = L_{total,2} - L_{total,1}$ as per figure S3 D. The stiffness k_{arm} of the arms was determined by applying a motor displacement on the tensile testing setup with a quasi non-deformable Dyneema SK78 strand of rope (*Metaltis*) and measuring the force. This resulted in a stiffness of k_{arm} = 20 N/mm of the arms, which was in turn used to calculate the estimated deformation curves of the tested samples via equation [1], corroborating the trends reported in the main text.

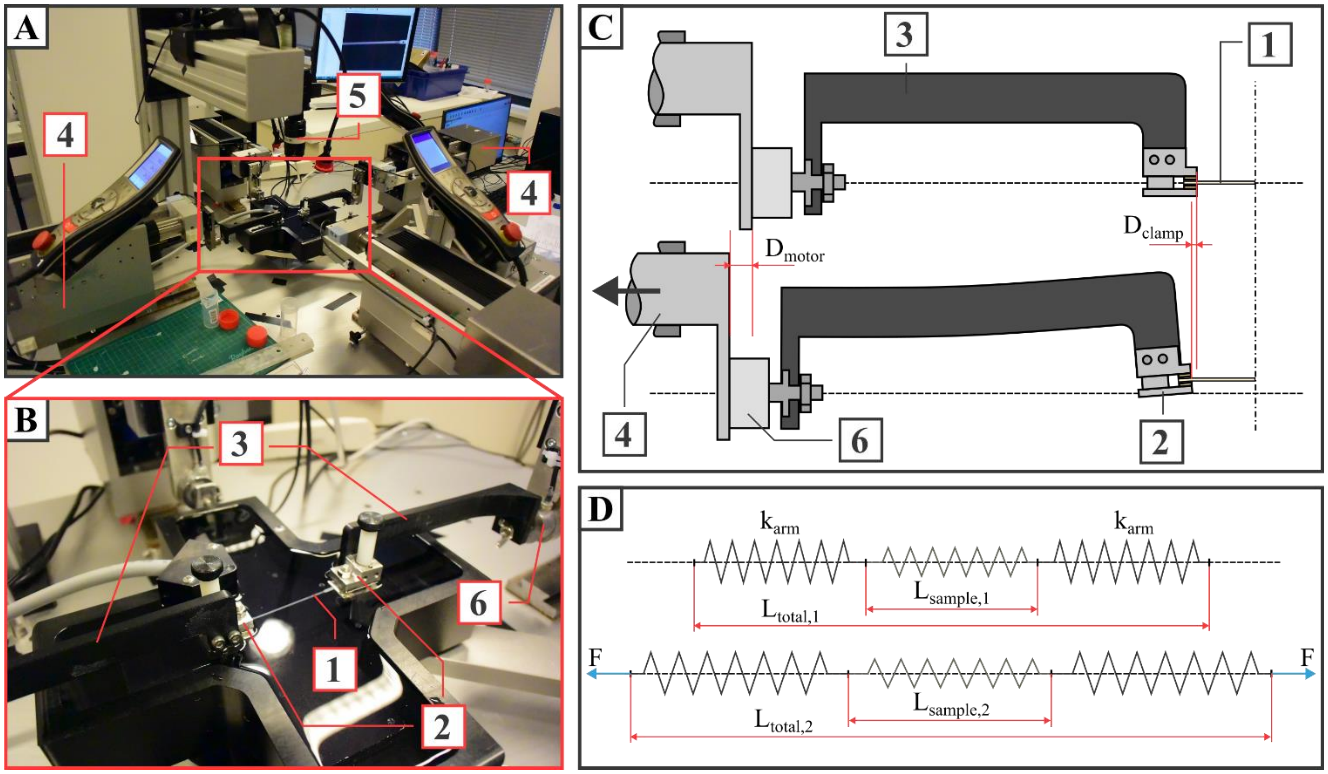

Figure S3: (A) Overview of the tensile testing setup with (B) a more detailed view and (C) a schematic overview of the tensile step. The sample (1) is fixated using two clamps (2), connected via 3D-printed arms (3) to the linear motors (4). Data measurements are done by an optical system (5), load cells (6) connected to the motors and linear encoders inside the motors (4). The compliance of the 3D-printed arms is depicted in (C) and the linearized model used for calculating the sample's deformation via encoder data is illustrated in (D).

D. Validation of viscoelastic parameters in Abaqus

As specified in the main text, we described this behaviour using a two-term Prony expansion of the dimensionless relaxation modulus $g_R(t)$ (1):

$$g_R(t) = 1 - \sum_{i=1}^{2} g_i \left(1 - e^{-\frac{t}{\tau_i}}\right), \qquad [2]$$

With g_i and τ_i being fitting parameters.

To confirm the practical validity of the fitted parameters to the Prony-expansion of the viscoelastic relaxation derived for all samples, this relaxation step was simulated in Abaqus for a non-degraded PCL sample. The sample was modeled according to the dimensions from figure S1 and meshed using hexagonal mesh. The following material parameters were used:

- Mass density: 1145 kg/m³;
- Elastic: isotropic:
  - Young's Modulus: 430 MPa;
  - Poisson's ratio: 0.22;
- Viscoelastic: time-domain Prony-expansion:
  - G1: 0.123, k1: 0, τ1: 0.814;
  - G2: 0.182, k2: 0, τ2: 9.15.

These parameters were obtained from the tensile tests and calculations described in the main text. Further, one end of the simulated dogbone sample was fixated, while a similar displacement load to the experimental case was imposed on the other. The relaxation hold was sustained for a time of 20 seconds, similar to the experimental case and force and deformation in the reduced section were monitored. Figure S4 shows the comparison of the tensile modulus between the experimental and the simulated case. These relaxation curves show a similar behavior, confirming the practical use of the obtained viscoelastic parameters.

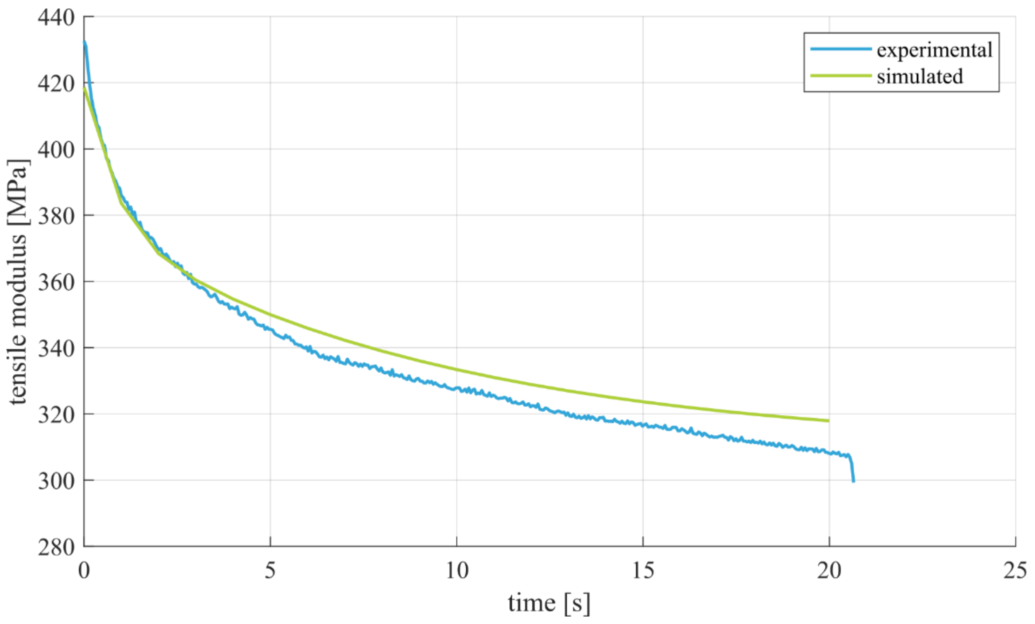

Figure S4: Comparison between the experimental and simulated tensile modulus of non-degraded PCL sample 1 during the first holding period. The modulus decreases during the relaxation, with similar behavior between the experimental and simulated case.

E. Enhanced graphs.

Figure S5 A and B display the stress-strain diagrams of non-degraded PCL and PLCL during the final pull, on which the point of modulus measurement have been indicated. The stress-strain diagram of PCL shows the clear yield point, followed by a sudden drop and a gradual of the measured stress. This behavior is not observed in PLCL samples, where no necking is seen, but rather a gradual stretching of the sample's reduced section. Figure S5 C and D show the evolution of the elasticity modulus of PCL and PLCL during the first ten days, clarifying the cluttered figure in the main text.

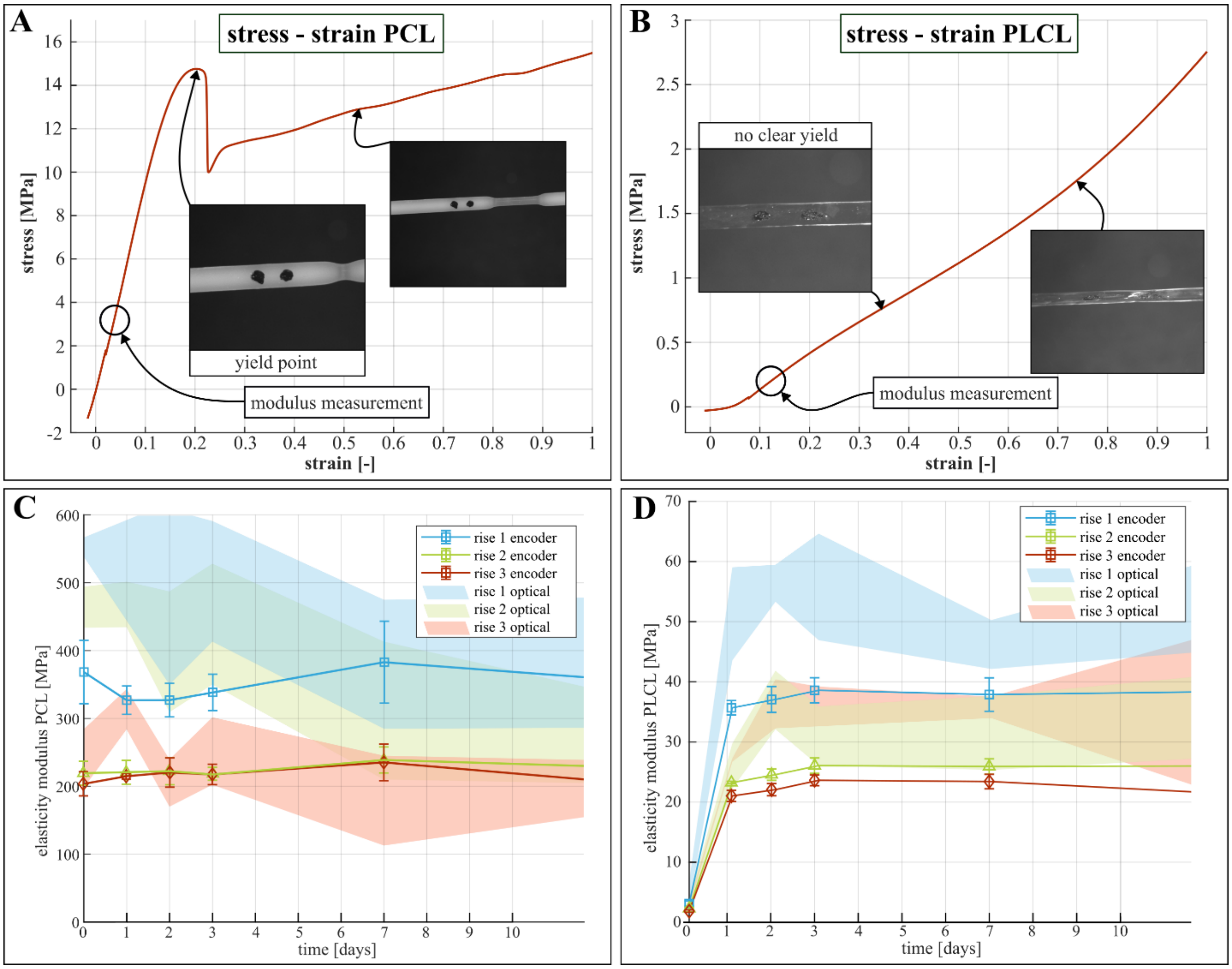

Figure S5: Enhanced graphs of tensile testing PCL and PLCL. (A & B) stress-strain diagram of non-degraded PCL and PLCL, (C & D) evolution of tensile modulus of PCL and PLCL during the first 10 days of degradation.

S2. Data used for analysis

This section tabulates the data points used to compose the figures in the main text and in SI.

Table S1: Yield stress and elongation at break for PCL and PLCL after different degradation times.

	Yield Stress *[MPa]*		Elongation at Break *[%]*	
degradation time *[days]*	PCL	PLCL	PCL	PLCL
0	14.5 ±0.2	9.5 ±2.3	>539 ±1	>651 ±0
1	14.5 ±0.2	3.8 ±0.1	>538 ±0	>586 ±41
2	14.4 ±0.2	4.1 ±0.1	>538 ±0	>573 ±53
3	14.6 ±0.1	4.4 ±0.2	>538 ±0	>626 ±23
7	14.6 ±0.1	4.6 ±0.1	>532 ±12	>616 ±47
14	14.2 ±0.8	4.6 ±0.2	402 ±196	529 ±124
21	13.8 ±0.5	4.1 ±0.2	21 ±4	106 ±19
28	10.6 ±0.7	3.8 ±0.1	11 ±0	57 ±10
35	11.4 ±1.1	3.8 ±0.1	11 ±2	49 ±2
42	11.0 ±1.0	3.7 ±0.2	12 ±2	41 ±8
56	8.2 ±1.2	1.4 ±0.1	8 ±2	6 ±1
70	5.5 ±0.9	1.1 ±0.3	6 ±1	9 ±6

Table S2: moduli of elasticity for PCL and PLCL after different degradation times. For each time, the moduli from optical data and calculated from encoder data are depicted.

		Young's modulus PCL *[MPa]*			Young's modulus PLCL *[MPa]*		
method	subm. time *[days]*	*rise 1*	*rise 2*	*rise 3*	*rise 1*	*rise 2*	*rise 3*
optical data	0	554.3 ±14.0	455.8 ±30.2	244.1 ±43.1	1.3 ±1.9	2.6 ±0.7	2.6 ±0.5
	1	504.1 ±50.9	459.8 ±33.5	316.7 ±29.6	50.8 ±7.7	25.5 ±3.7	26.5 ±0.3
	2	482.3 ±133.4	390.0 ±114.4	207 ±34.7	55.9 ±3.1	36.5 ±2.5	35.9 ±4.1
	3	503.8 ±88.8	431.0 ±88.9	255.2 ±49.7	55.3 ±8.9	30.5 ±4.9	35.4 ±3.3
	7	381.7 ±95.0	303.4 ±101.5	182.0 ±66.1	45.7 ±4.1	31.0 ±6.2	35.3 ±1.8
	14	391.5 ±90.0	249.8 ±55.7	208.7 ±30.1	54.6 ±8.8	34.8 ±7.1	34.1 ±17.2
	21	429.6 ±92.0	343.0 ±99.8	213.2 ±17.1	40.0 ±22.7	47.7 ±2.2	39.3 ±3.2
	28	315.9 ±25.1	261.2 ±75.8	399.8 ±130.6	53.9 ±9.6	35.1 ±9.0	32.8 ±8.6
	35	329.1 ±34.0	340.9 ±76.4	325.6 ±33.8	61.0 ±7.6	36.5 ±2.0	26.5 ±5.0
	42	414.3 ±49.7	464.1 ±93.4	447.7 ±92.2	52.0 ±20.8	23.5 ±6.6	17.2 ±2.0
	56	376.9 ±42.2	336.1 ±43.0	321.9 ±18.1	41.8 ±7.1	11.4 -	- -
	70	355.3 ±101.6	383.1 -	381.5 -	40.8 ±9.7	- -	- -
encoder data	0	368.4 ±46.6	219.3 ±17.6	203.6 ±17.8	3.1 ±0.4	2.3 ±0.2	1.9 ±0.2
	1	326.9 ±21.0	220.6 ±17.6	214.8 ±4.3	35.7 ±1.2	23.2 ±0.2	21.0 ±0.9
	2	326.9 ±24.7	222.3 ±20.1	220.3 ±21.6	37.1 ±2.2	24.6 ±1.0	22.1 ±1.0
	3	336.5 ±26.7	218.4 ±9.5	217.4 ±15.1	38.6 ±2.1	26.1 ±1.3	23.6 ±0.9
	7	382.8 ±60.4	238.7 ±19.2	235.2 ±27.0	37.9 ±2.8	25.9 ±1.3	23.4 ±1.2
	14	349.6 ±61.2	225.5 ±22.1	197.7 ±20.7	38.5 ±4.4	26.0 ±1.5	20.8 ±2.6
	21	339.7 ±26.2	234.9 ±26.6	212.8 ±15.8	36.1 ±6.1	23.3 ±3.3	20.8 ±2.8
	28	302.7 ±26.2	243.3 ±24.3	233.2 ±14.0	40.7 ±1.6	24.6 ±0.4	21.9 ±0.9
	35	319.2 ±21.5	247.6 ±16.4	241.9 ±13.7	42.3 ±1.3	25.4 ±0.7	22.2 ±0.6
	42	326.3 ±31.0	245.3 ±19.3	235.4 ±20.0	37.9 ±3.3	23.5 ±2.5	20.4 ±2.3
	56	334.1 ±17.7	249.8 ±12.3	233.6 ±12.6	32.5 ±5.0	14.3 -	- -
	70	344.7 ±9.0	262.7 -	241.1 -	20.5 ±8.8	- -	- -

Table S3: Viscoelastic Prony coefficients (see eq. [2]) in function of degradation time for PCL and PLCL.

	PCL				PLCL			
days	g_1 *[-]*	τ_1 *[s]*	g_2 *[-]*	τ_2 *[s]*	g_1 *[-]*	τ_1 *[s]*	g_2 *[-]*	τ_2 *[s]*
0	0.11 ±0.01	0.89 ±0.06	0.18 ±0.02	9.05 ±0.81	0.16 ±0.02	2.00 ±0.21	0.34 ±0.01	25.14 ±1.53
1	0.09 ±0.01	0.90 ±0.011	0.14 ±0.01	8.10 ±1.10	0.14 ±0.01	1.30 ±0.18	0.16 ±0.00	20.16 ±3.01
2	0.10 ±0.02	0.91 ±0.16	0.14 ±0.01	10.41 ±3.23	0.13 ±0.01	1.39 ±0.21	0.15 ±0.01	19.01 ±1.51
3	0.10 ±0.01	0.90 ±0.22	0.15 ±0.01	9.60 ±1.97	0.14±0.02	1.16 ±0.11	0.16 ±0.01	16.98 ±0.52
7	0.09 ±0.01	0.84 ±0.10	0.15 ±0.01	8.47 ±1.39	0.14 ±0.01	1.13 ±0.11	0.16 ±0.01	18.22 ±1.06
14	0.09 ±0.01	1.00 ±0.17	0.16 ±0.01	11.50 ±1.80	0.13 ±0.01	1.31 ±0.24	0.17 ±0.01	18.67 ±2.32
21	0.07 ±0.01	1.07 ±0.24	0.17 ±0.06	14.50 ±11.36	0.14 ±0.01	1.23 ±0.13	0.19 ±0.00	19.01±3.08
28	0.07 ±0.01	0.75 ±0.23	0.15 ±0.05	14.53 ±15.01	0.15 ±0.01	1.42 ±0.18	0.18 ±0.01	18.62 ±1.81
35	0.08 ±0.02	0.72 ±0.22	0.13 ±0.02	8.52 ±1.56	0.16 ±0.01	1.35 ±0.14	0.18 ±0.01	18.39 ±1.50
42	0.09 ±0.02	0.94 ±0.21	0.13 ±0.02	11.13 ±3.68	0.16 ±0.01	1.30 ±0.21	0.19 ±0.01	18.23 ±2.42
56	0.11 ±0.01	1.06 ±0.14	0.14 ±0.01	11.57 ±2.12	- -	- -	- -	- -
70	0.10 -	1.06 -	0.16 -	12.20 -	- -	- -	- -	- -

References

1. *ABAQUS Analysis User's Manual 6.14, 22.7.1 Time domain viscoelasticity. Simulia, 2014.*
